# Unraveling the role and mechanism of mitochondria in postoperative cognitive dysfunction: a narrative review

**DOI:** 10.1186/s12974-024-03285-3

**Published:** 2024-11-12

**Authors:** Zhenyong Zhang, Wei Yang, Lanbo Wang, Chengyao Zhu, Shuyan Cui, Tian Wang, Xi Gu, Yang Liu, Peng Qiu

**Affiliations:** 1 Department of Oncology, Shengjing Hospital of China Medical University, Shenyang, 110004 Liaoning Province China; 2 Department of Infectious Disease, Shengjing Hospital of China Medical University, Shenyang, 110004 Liaoning Province China; 3 Department of Radiology, Shengjing Hospital of China Medical University, Shenyang, 110004 Liaoning Province China; 4 Department of Anesthesiology, Shengjing Hospital of China Medical University, Shenyang, 110004 Liaoning Province China

**Keywords:** Postoperative cognitive dysfunction, Cognitive complication, Mitochondria, Mitochondrial dysfunction, Oxidative stress, Mitophagy, Neuroinflammation, Electron transport chain deficiency

## Abstract

Postoperative cognitive dysfunction (POCD) is a frequent neurological complication encountered during the perioperative period with unclear mechanisms and no effective treatments. Recent research into the pathogenesis of POCD has primarily focused on neuroinflammation, oxidative stress, changes in neural synaptic plasticity and neurotransmitter imbalances. Given the high-energy metabolism of neurons and their critical dependency on mitochondria, mitochondrial dysfunction directly affects neuronal function. Additionally, as the primary organelles generating reactive oxygen species, mitochondria are closely linked to the pathological processes of neuroinflammation. Surgery and anesthesia can induce mitochondrial dysfunction, increase mitochondrial oxidative stress, and disrupt mitochondrial quality-control mechanisms via various pathways, hence serving as key initiators of the POCD pathological process. We conducted a review on the role and potential mechanisms of mitochondria in postoperative cognitive dysfunction by consulting relevant literature from the PubMed and EMBASE databases spanning the past 25 years. Our findings indicate that surgery and anesthesia can inhibit mitochondrial respiration, thereby reducing ATP production, decreasing mitochondrial membrane potential, promoting mitochondrial fission, inducing mitochondrial calcium buffering abnormalities and iron accumulation, inhibiting mitophagy, and increasing mitochondrial oxidative stress. Mitochondrial dysfunction and damage can ultimately lead to impaired neuronal function, abnormal synaptic transmission, impaired synthesis and release of neurotransmitters, and even neuronal death, resulting in cognitive dysfunction. Targeted mitochondrial therapies have shown positive outcomes, holding promise as a novel treatment for POCD.

## Introduction

Postoperative cognitive dysfunction (POCD) is a frequent complication of the central nervous system (CNS) after surgical anesthesia and is especially prevalent among older patients [1, 2]. The underlying mechanisms of POCD are complex and still not fully understood, but recent advancements have been made in research and treatment. The possible mechanisms of POCD include neuroinflammation, oxidative stress, neurotransmitter imbalances, and changes in neural synaptic plasticity [3]. Additionally, the occurrence of POCD is influenced by various factors such as age, preoperative cognitive status, type of surgery, anesthesia method, and postoperative pain [1]. Effective prevention and treatment methods for POCD are still lacking.

Based on the current research findings regarding the mechanism of POCD, surgery and anesthesia may trigger neuronal death via mechanisms like neuroinflammation and oxidative stress, ultimately resulting in cognitive impairment [4]. Anesthetic drugs can disrupt synaptic connections and communication among neurons, causing a maladjustment within the neuronal network, thereby affecting memory and cognitive abilities [5]. Neuroinflammation can also compromise the stability and plasticity of synaptic connections, leading to communication barriers between neurons [3]. Furthermore, certain anesthetic drugs may alter the activity of the brain's enzyme system, impacting the synthesis and metabolism of neurotransmitters, which could potentially exert adverse effects on cognitive function [6, 7]. Throughout these pathological processes, mitochondria are involved and exert a crucial regulatory function.

Mitochondria, known as the “energy factories” of cells, play a crucial role in the respiratory chain and in ATP production; they are abundantly present in nervous system cells [8]. Given the specific nature of neuronal function, which requires a large amount of energy, there is a great dependence on mitochondria [8]. Factors such as surgical trauma, anesthetic drugs, and postoperative stress may interfere with the normal function of mitochondria, resulting in mitochondrial respiratory chain blockage, mitochondrial membrane potential decline, cytochrome C release, mitochondrial ion homeostasis imbalance, and mitochondrial dynamics abnormalities [6, 9–13]. These disruptions can lead to insufficient energy supply to neurons, potentially causing neuronal death and subsequently affecting the cognitive function of the brain [9]. The oxidative stress response generated during surgery and anesthesia can produce a large amount of free radicals and reactive oxygen species (ROS), leading to mitochondrial damage. Damaged mitochondria not only fail to effectively synthesize ATP but also further release ROS, thus forming a vicious cycle [14]. Synaptic transmission in neurons demands significant energy support [15, 16]. Mitochondrial dysfunction and ATP production deficiencies, resulting from anesthesia and surgery, can disrupt normal synaptic transmission and function [17]. Calcium ions play a pivotal regulatory role in neuronal synaptic connections [18]. Mitochondria are instrumental in regulating intracellular calcium concentration, thereby ensuring the smooth operation of synaptic transmission [18]. An imbalance in mitochondrial calcium homeostasis due to anesthesia and surgery may cause abnormalities in neuronal synaptic connections [6]. During brain development, general anesthesia can cause long-term impairments in inhibitory synaptic transmission [5]. Mitochondrial damage serves as a significant driver of neuroinflammation, ultimately leading to neuronal death and synaptic dysfunction [19].

The synthesis, release, and reuptake of neurotransmitters also rely on mitochondrial energy supply and the regulation of ion homeostasis and redox status [20]. Additionally, quality control mechanisms, such as mitophagy, are essential for preserving normal mitochondrial function. Anesthesia and surgery-induced abnormalities in mitophagy constitute one of the potential factors contributing to POCD [21].

Hence, mitochondria may significantly influence the pathological process of POCD. A thorough understanding of mitochondrial mechanisms is crucial to prevent and treat POCD. This article reviews the current research on the role and mechanisms of mitochondria in the development of POCD.

## Methods

As a narrative review, we conducted a literature search in the PubMed and EMBASE databases. The search keywords included: Mitochondria, Mitochondrial Dysfunction, Mitochondrial Respiratory Chain Deficiency, Oxidative Phosphorylation Deficiency, Electron Transport Chain Deficiency, Oxidative Stress, mitochondrial autophagy, mitophagy, Cognitive Dysfunction, Postoperative Cognitive Dysfunction, Postoperative Cognitive Complication, and Postoperative Cognitive Disorders. The search covered the period from 2000 to 2025, in English, and included both human and animal studies. We conducted a relevance screening of the search results and also performed a supplementary search for valuable references in the included literature.

## The effects of anesthesia and surgery on mitochondrial function

Mitochondrial dysfunction is linked to the early pathogenesis of cognitive impairment caused by general anesthesia in both developing and aging mammalian brains [5, 22]. Mitochondria may be an important early target of neuronal development and synaptic injury induced by general anesthesia [13].

**Fig. 1:**
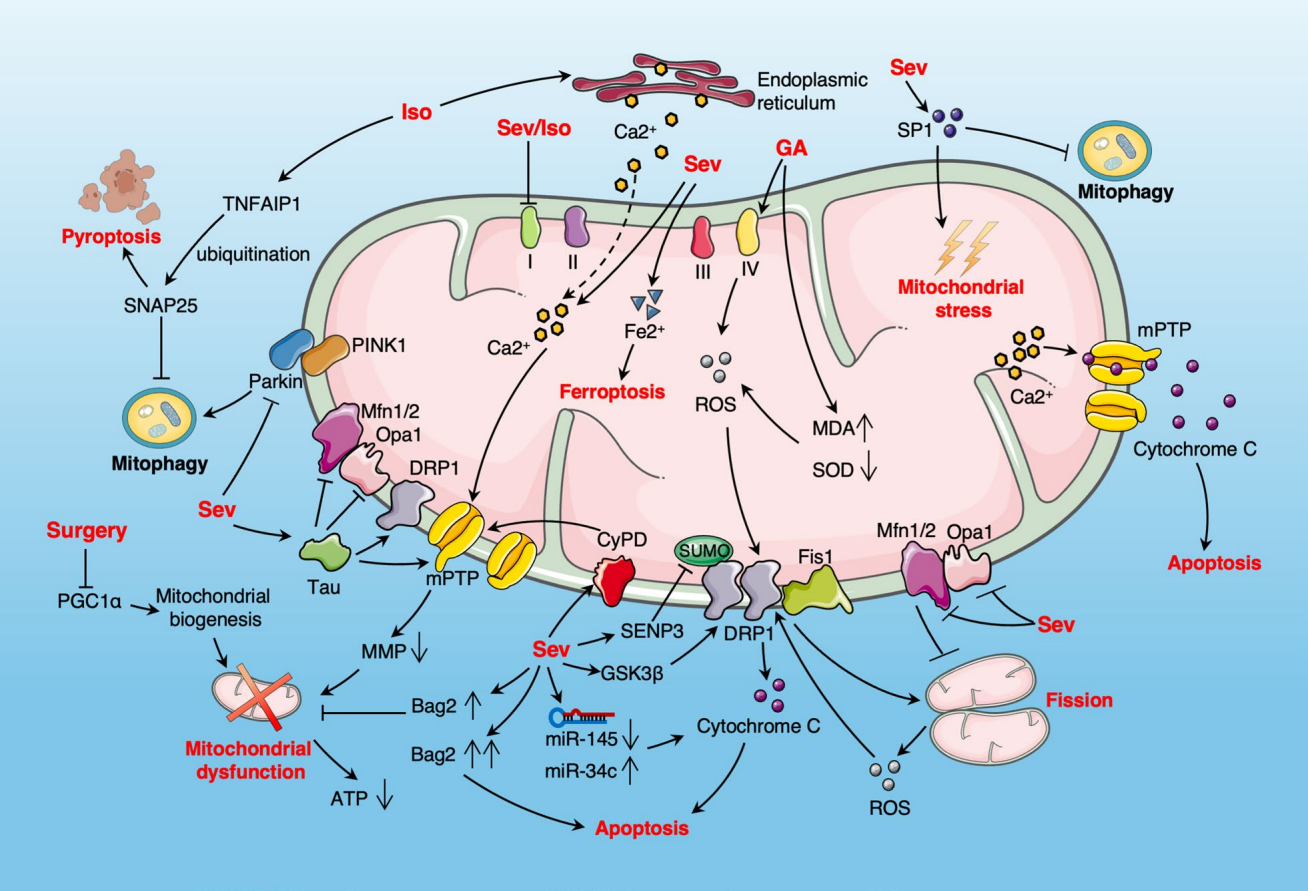
The impact of surgery and anesthesia on mitochondrial function. Surgery and anesthesia, especially general anesthesia, can have extensive effects on mitochondrial function during the perioperative period. Surgery and anesthesia can lead to increased levels of mitochondrial oxidative stress and further generation of ROS by promoting mitochondrial fission, forming a vicious cycle of oxidative stress. General anesthetic drugs can inhibit mitochondrial respiration and interfere with ATP production. Surgery and anesthesia can lead to mitochondrial calcium overload and iron homeostasis imbalance, and cause cell death (apoptosis and ferroptosis) through a series of downstream mechanisms. Surgery and anesthesia can affect mitochondrial biogenesis and dynamics (transport, fusion, fission), and interfere with important mitochondrial quality control mechanisms such as the unfolded protein response and mitophagy, leading to mitochondrial dysfunction. In addition, general anesthesia can also affect mitochondrial dynamics and mitochondrial function by increasing the phosphorylation of Tau protein

Anesthesia and surgery lead to increased levels of mitochondrial oxidative stress by increasing malondialdehyde (MDA) activity and decreasing superoxide dismutase (SOD) activity [13, 23]. Studies have confirmed that volatile anesthetics, as well as pentobarbital and propofol, can dose-dependently inhibit mitochondrial respiration [9, 24] (Fig. 1). Isoflurane and sevoflurane selectively inhibit the respiratory chain complex I, and nitrous oxide inhibits complex IV [25]. Combined anesthesia with isoflurane, nitrous oxide, and midazolam induces mitochondrial swelling, impaired structural integrity, increased complex IV activity, and reduced distribution in the presynaptic neuronal distribution area in rats [5]. General anesthesia enhances complex IV activity while reducing mitochondrial SOD activity, thereby leading to excessive ROS production. This promotes mitochondrial fission and increases ROS generation, creating a vicious cycle of oxidative stress [13, 26, 27] (Fig. 1). In addition, anesthesia and surgery can lead to mitochondrial calcium overload [28, 29] and iron homeostasis imbalance [30, 31], resulting in mitochondrial dysfunction (Fig. 1). In developing rats, general anesthesia induces excessive mitochondrial fission in the brain, promoting leakage of cytochrome C and subsequent neuronal apoptosis [5, 13]. General anesthesia also affects mitochondrial quality control mechanisms, including the mitochondrial unfolded protein response [32].

Research has found that volatile anesthetics such as sevoflurane and isoflurane have the most significant impact on mitochondria (Fig. 1; Table 1). Sevoflurane increases neurotoxicity and reduces cognitive function in aged rodents by impairing mitochondrial dynamics, inducing mitochondrial dysfunction, and promoting cell apoptosis [33–35]. Sevoflurane can lead to a decrease in mitochondrial density in rat brain tissue [13, 36] and induce neurotoxicity in neonatal mice by promoting GSK3β/drp1-dependent mitochondrial fission [33]. Sevoflurane can induce the upregulation of specific protein 1 (SP1) in POCD animal models; interfering with SP1 can significantly inhibit sevoflurane-induced oxidative stress and mitochondrial dysfunction [37]. Additionally, sevoflurane downregulates the expression of miR-145 in hippocampal neurons [38] and upregulates the expression of miR-34c [39], thereby inducing apoptosis through the mitochondrial pathway. Sevoflurane also upregulates CypD expression, impairing mitochondrial function and leading to cognitive impairment in mice [40]. Moreover, it increases Tau protein phosphorylation [40, 41], which in its hyperphosphorylated state, disrupts mitochondrial transport, dynamics, and permeability [42], thereby diminishing mitochondrial metabolism [41]. In addition, exposure to sevoflurane can inhibit hippocampal mitophagy, exacerbating mitochondrial dysfunction and neuroinflammation [43]. Sevoflurane can also cause an imbalance in mitochondrial iron and calcium homeostasis [28, 30, 31]. Mitochondrial lipid peroxidation showed an increase in neonatal mice following treatment with sevoflurane, which leads to mitochondrial iron accumulation and neuronal ferroptosis, resulting in cognitive deficits [30, 31]. Sevoflurane induces mitochondrial calcium overload by activating inositol 1,4,5-trisphosphate (IP3) and other receptors on the endoplasmic reticulum (ER) membrane, leading to the opening of mitochondrial permeability transition pore (mPTP) and loss of mitochondrial membrane potential (MMP) [28]. In addition to its effects on neuronal mitochondria, sevoflurane also reduces mitochondrial function in microglia, leading to a decrease in ATP production and an inability of the microglia to effectively clear damaged neurons [44]. When newborns rats are exposed to sevoflurane, long-term ultrastructural damage such as reduced presynaptic mitochondrial localization may occur [45]. Repeated exposure to sevoflurane can lead to mitochondrial dysfunction and reduced ATP production in the brain tissue of young mice [46]. Sevoflurane exposure significantly increases Bag2 protein levels in a time- and dose-dependent manner [47]. The Bag family of proteins inhibit cell death through interactions with Bcl-2. Bag2 participates in protein folding and proteasome degradation pathways and promotes mitochondrial autophagy via interaction with PINK1 [47]. Bag2 can alleviate the decrease in MMP and ATP production caused by sevoflurane exposure, which is considered a stress-protective mechanism of mitochondria after sevoflurane exposure [47]. However, excessive elevation of Bag2 could lead to its neuroprotective effect turning into neurotoxicity.

**Table 1:** Negative effects of volatile anesthetics on mitochondria

Volatile anesthetics	Effects on mitochondria	References
Sevoflurane	Mitochondrial dynamics impaired (decreased transportation and metabolism, increased division)	[33, 41, 42]
	Decreased mitochondrial density	[13, 36]
	Mitochondrial dysfunction	[37, 40, 46]
	Mitochondria-mediated apoptosis	[38, 39]
	Increased mitochondrial oxidative stress	[37]
	Decreased mitophagy	[43]
	Mitochondrial calcium overload	[28, 29]
	Mitochondrial iron homeostasis imbalance	[30, 31]
	Mitochondrial respiratory chain obstruction	[25]
Isoflurane	Mitochondrial swelling and vacuolar formation	[48]
	Mitochondrial respiratory chain obstruction	[5, 25, 28]
	Mitochondrial dysfunction	[9, 28]
	Mitochondrial calcium overload	[51]
	Decreased mitophagy	[52]

Mitochondrial dysfunction is an early trigger of isoflurane-induced neuronal damage [48]. Isoflurane can lead to mitochondrial swelling and vacuolization, MMP decline, and electron transport chain dysfunction in the rat hippocampus, resulting in reduced ATP production, oxidative damage to neurons, and neuronal apoptosis, all of which can ultimately cause POCD [49, 50]. Extended isoflurane exposure triggers excessive calcium release from the ER, depleting its calcium stores and causing mitochondrial calcium overload, which can be cytotoxic to neurons [51]. Isoflurane and surgery can induce the activation of ubiquitin ligase TNFAIP1 in the hippocampus, which inhibits mitochondrial autophagy and promotes neuronal pyroptosis through ubiquitination of synapse-associated protein 25 (SNAP25) [52].

## Mitochondrial dysfunction’s impact on POCD

### The importance of mitochondrial dysfunction in the occurrence of POCD

Mitochondria are dynamic organelles that continuously undergo fission and fusion, processes essential for energy metabolism, calcium buffering, and cell survival regulation. Densely distributed in the CNS, mitochondria provide essential energy for neurons and influence synaptic plasticity [53, 54]. Mitochondria are abundant in the distal regions of neurons, particularly prone to dysfunction because of the high energy demands of synapses [55], leading to CNS diseases such as cognitive disorders [53]. Mitochondrial dysfunction is considered one of the early pathogenic factors of cognitive impairment in the developing or aging brain after general anesthesia [22, 56], and it is also a significant feature of aging and neuronal degeneration [22, 23]. The vicious cycle of reduced mitochondrial function and increased ROS generation has been shown to be associated with the pathogenesis of POCD [14]. Mitochondrial damage, characterized by morphological changes, reduced membrane potential, and disrupted electron transport chain (ETC) complex function, is a key factor in the pathogenesis of POCD [57]. Metabolomic analysis indicates that POCD is linked to disruptions in several signaling pathways such as the nitric oxide, PI3K-AKT, mTOR, mitochondrial dysfunction, and NF-κB pathways [58].

Numerous studies have confirmed the genetic association between mitochondria and POCD. Sharpley et al. reported the impact of mitochondrial gene variations on cognitive function in mice [59]. In older subjects, the heteroplasmy rate of specific mutation sites in respiratory chain complex I is linked to cognitive decline as measured by the modified Mini-Mental State Examination scores [60]. Mutations in mitochondrial DNA (mtDNA) exceeding a certain threshold may affect cognitive performance. Mitochondria depend on nuclear genes for normal function, and polymorphisms in these genes may cause mild cognitive impairment [53]. Reduced levels of the *TOMM40* gene, which encodes the translocase of outer membrane (TOM) subunit, can cause mitochondrial dysfunction and is independently associated with cognitive decline [61]. Abnormalities in genes encoding mitochondrial monofunctional 10-formyltetrahydrofolate synthetase (C1-THF synthase/*MTHFD1L*) [62], vacuolar ATPase-related *ATP6V1B2* [63], and monoamine oxidase (*MAO*) [64] lead to mitochondrial dysfunction and are associated with depression and cognitive impairment. Functional annotation and DE-lncRNA-mRNA co-expression networks indicate that DE-lncRNAs are linked to mitochondrial dysfunction, oxidative stress during sevoflurane anesthesia, age-related metabolic changes, DNA damage, apoptosis, and neurodegenerative traits [65, 66]. Nfe2l2, Mthfd1l, Akt1, and Prkcd are targets of DE-lncRNAs in metabolic pathways, influencing mitochondrial autophagy, membrane potential, and apoptosis [65, 66].

Mitochondrial quality control (QC) comprises the mitochondrial unfolded protein response (mtUPR), ubiquitin–proteasome system (UPS), mitochondrial-derived vesicle (MDV) degradation pathway, and mitophagy and is essential for normal neuronal function [67–70]. Dysfunctional mitochondrial QC pathways can adversely affect cells such as neurons that depend heavily on mitochondria [42]. Anesthesia and surgery have been proven to have extensive effects on mitochondrial QC mechanisms such as mtUPR, particularly mitophagy [21, 25, 31], which will be discussed in detail below.

### Mitochondrial respiratory chain obstruction

Volatile general anesthetics as well as pentobarbital and propofol have been found to inhibit mitochondrial respiration in a dose-dependent manner [9, 10]. Mitochondria are particularly sensitive to volatile anesthetics; the effects of intravenous anesthetics are relatively small [9]. Anesthetics have a dual effect on the mitochondrial respiratory chain complex. The inhibitory effect of anesthetics on mitochondrial respiration helps the drugs exert their anesthetic effects [71, 72]; however, it may induce postoperative delirium and POCD in older patients, as well as neuronal apoptosis in developing brains [73]. The negative impacts of anesthesia on mitochondrial respiratory chain are often time- and dose-dependent [5, 9, 10, 13, 14]. The conventional dosage of anesthetics used in clinical practice only affects individuals with high sensitivity and poor tolerance, such as older patients and children in the developmental stage of the nervous system [5, 13, 14]. This also explains why these populations are prone to POCD.

Isoflurane exposure in rats significantly increased the activity of mitochondrial respiratory chain complexes I and II, while inhibiting complex IV (cytochrome C oxidase) activity [49]. Similarly, sevoflurane and nitrous oxide can also inhibit complex IV in rats [10, 25, 74]. The decreased activity of complex IV impedes ATP production and also leads to an increase in Ca^2+^ -independent glutamate release, triggering neuronal excitotoxic cell death [49]. Volatile anesthetics have been shown to inhibit complex I in a dose-dependent manner [9, 10]. Sevoflurane inhibits complex I activity by upregulating calmodulin-dependent protein kinase II (CaMKII) expression and decreasing NAD+ production [44]. Halothane enhances cytochrome C release [9]. High-concentration and prolonged use of pentobarbital and propofol can inhibit complex I [10, 24]; propofol is currently the only known general anesthetic drug that reduces mitochondrial respiratory chain complex II [9]. Oxygen consumption rate (OCR) is a key measure of mitochondrial respiratory capacity. Sevoflurane was found to attenuate the reduction in OCR and maximum respiration associated with ATP production in mouse cerebral vascular endothelial cells [35]. Coenzyme Q (CoQ) is essential for the mitochondrial respiratory chain, as it facilitates electron transfer from complexes I and II to complex III. Research has shown that CoQ10 can reduce cognitive deficits induced by sevoflurane in mice [46], suggesting that sevoflurane may trigger POCD by affecting mitochondrial respiration.

The inhibitory effect of volatile anesthetics on complex I may negatively affect mitochondrial energy production in the nervous system. However, in certain pathological processes of ischemia–reperfusion injury, such as myocardial ischemia and stroke, this inhibition may exert a protective effect [75, 76]. Studies have found that the mitochondrial respiratory chain complex I inhibitor rotenone can alleviate the damage caused by cerebral ischemia by inhibiting the opening of mPTP and the generation of ROS [76]. Volatile anesthetics at clinical concentrations inhibit complex I, leading to a decrease in presynaptic MMP and reducing Ca^2+^ overload in presynaptic terminals in ischemic regions, thereby mitigating apoptosis, necrosis, and oxidative stress [10]. Similarly, the inhibitory effect of clinical concentrations of volatile anesthetics on the respiratory chain has also been demonstrated not to impair cardiac function, and may instead serving as a potential mechanism for the protective effect of volatile anesthetics preconditioning on myocardial ischemia [25]. The inhibition of complex I activity by volatile anesthetics reduces the reverse electron transport and the subsequent generation of large amounts of ROS driven by mitochondria during myocardial and cerebral ischemia–reperfusion [75, 77].

### Mitochondrial dynamic abnormalities

Maintaining a balance between mitochondrial fission and fusion is essential for intracellular homeostasis [78]. Mitochondrial fission allows for mitochondrial renewal and redistribution to synapses, while mitochondrial fusion supports mitochondrial protein regeneration, DNA repair, and functional recovery [78, 79]. The GTPases Mfn1 and Mfn2 control mitochondrial outer membrane fusion [80], while Opa1 controls inner membrane fusion [81]. Mitochondrial fission is primarily regulated by fission 1 (Fis1) and dynamin-related protein 1 (Drp1) [82]. Drp1 is a crucial regulator of mitochondrial fission and significantly influences neurite development and synapse formation [83]. Drp1 activation is controlled by other upstream processes such as MAPK/ERK activation [84]. Drp1 is activated by various cellular stimuli, translocates from the cytoplasm to the outer mitochondrial membrane, interacts with Fis1, and induces mitochondrial fission [82].

Research indicates that continuous mitochondrial fission can lead to mitochondrial dysfunction and excessive production of ROS, ultimately resulting in neuronal death [85]. General anesthesia increases ROS production by reducing SOD activity and promotes excessive mitochondrial fission, leading to mitochondrial morphological disorders [13]. Mitochondria with excessive fission function poorly and are more likely to produce more ROS, thereby forming a vicious cycle [13] (Fig. 1). In addition, mitochondrial fission induced by general anesthesia may also promote acute leakage of cytochrome C, leading to activation of apoptosis through the mitochondrial pathway [13]. Research indicates that sevoflurane alters mitochondrial morphology and induces neuronal damage by promoting mitochondrial fission and inhibiting fusion. This is achieved through the upregulation of Drp1 and Fis1 and the downregulation of Opa1 and Mfn1/2 expression [34, 86, 87] (Fig. 1). Pretreatment with the Drp1-selective inhibitor—Mdivi-1—can protect mitochondrial function and reduce synaptic damage and neuronal toxicity [88]. Sevoflurane can lead to increased phosphorylation of Tau protein [40], which, through the interaction of Tau protein with related proteins such as Drp1, can lead to increased mitochondrial fission and decreased fusion, ultimately resulting in synaptic damage and cognitive impairment [42, 89, 90] (Fig. 1). Sevoflurane induces excessive mitochondrial fission by activating the GSK-3β pathway, which mediates Drp-1 phosphorylation at ser616 [33] (Fig. 1).

SUMOylation is a post-translational modification involving the covalent attachment of small ubiquitin-like modifier (SUMO) proteins to target proteins, influencing their function and localization [91]. SUMOylation of Drp1 is crucial for mitochondrial function maintenance [92, 93]. Research indicates that sevoflurane elevates SUMO-specific protease 3 (SENP3) expression in the hippocampus of older individuals, resulting in Drp1 deSUMOylation and excessive mitochondrial fission [87] (Fig. 1).

Mitochondrial dysfunction primarily arises from the dysregulation of mitochondrial biogenesis [94, 95]. PGC-1α plays a crucial role in regulating mitochondrial biogenesis and cellular metabolism [96, 97]. Studies have found that surgery has a negative effect on mitochondrial biogenesis, while activation of the PGC-1α/BDNF pathway can improve mitochondrial health and reduce perioperative neurocognitive impairment [98] (Fig. 1).

### Decreased MMP

During respiratory oxidation, mitochondria convert generated energy into electrochemical potential energy within their inner membrane, resulting in an asymmetric distribution of protons and ions that forms the MMP [99]. This MMP is considered a core indicator of mitochondrial function and is crucial for the synthesis of ATP and the maintenance of calcium homeostasis [6, 10, 100]. The decrease in MMP (i.e., depolarization of mitochondrial membrane) is of great significance in the early stages of mitochondrial damage [11]. Mitochondrial membrane depolarization is controlled by mechanisms including the opening of the mPTP, calcium ion influx, activation of the mitochondrial ATP-regulated potassium channel (mitoKATP), and alterations in mitochondrial respiratory chain complex functions. Studies have found that mitochondrial membrane depolarization induced by volatile anesthetics may be partially caused by the activation of mitoKATP [10, 74, 101]. Although intravenous anesthetics such as propofol and pentobarbital do not directly affect the opening of mitoKATP, they do inhibit the isoflurane-induced mitochondrial K+ influx [102]. Although studies have found that general anesthesia-induced mitochondrial depolarization is not directly related to Ca^2+^ influx [24, 74], mitochondrial calcium overload caused by Ca^2+^ influx can lead to a decrease in MMP through a series of mechanisms, which are discussed in subsequent sections of this review. The effect of sevoflurane on MMP may be related to the reversal of ATP synthase [10, 24]. Sevoflurane was shown to cause mitochondrial dysfunction by impairing MMP and enhancing the production of ROS in human neuronal SH-SY5Y cells [21, 47]. Isoflurane exposure can open the mPTP and decrease MMP, reducing ATP production and releasing cytochrome C into the cytosol, which triggers neuronal apoptosis [49, 50].

### Mitochondrial calcium overload

**Fig. 2:**
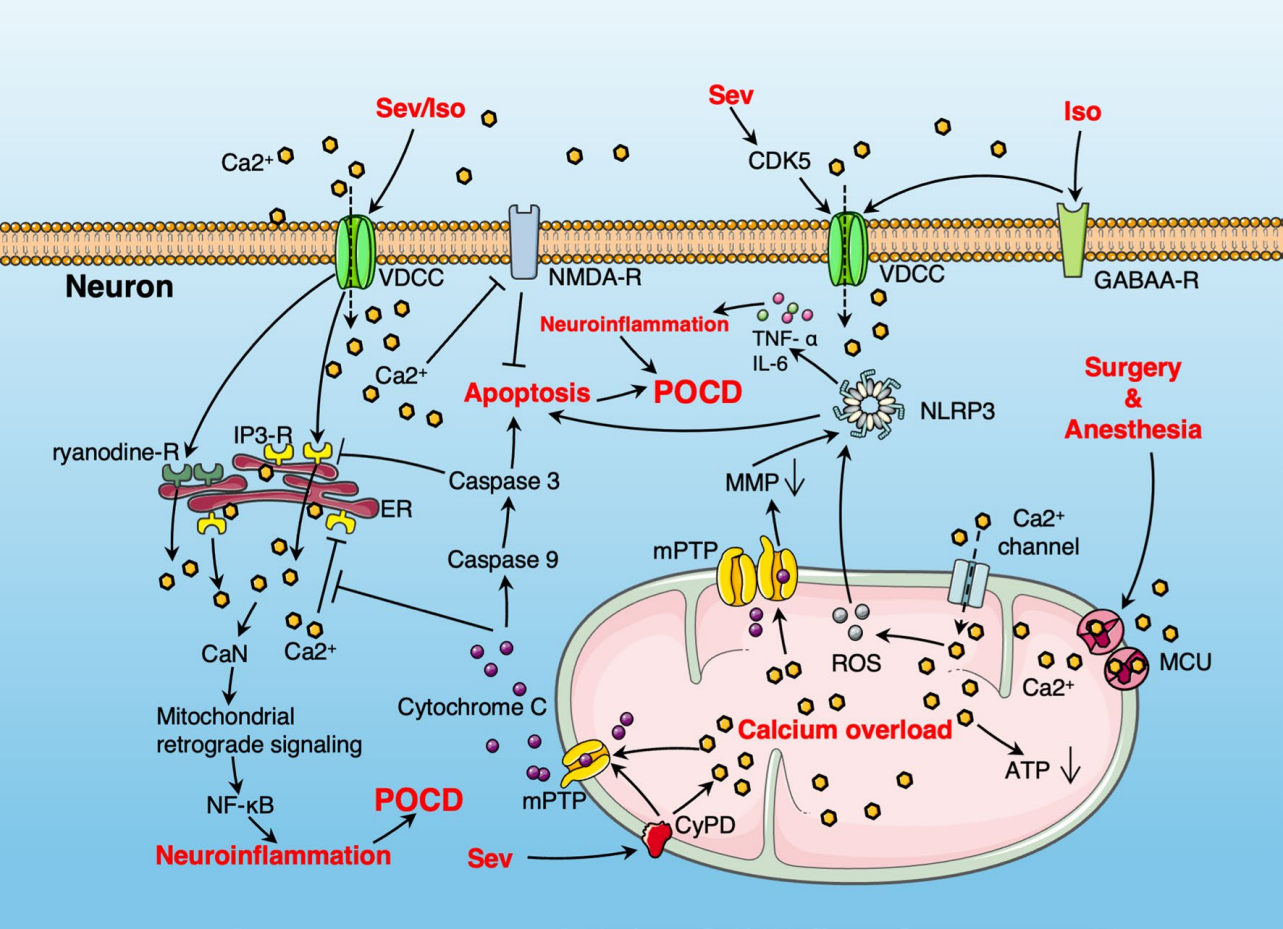
The role and mechanism of mitochondrial calcium overload in POCD. Anesthesia and surgery increase the calcium influx into neurons by opening voltage-dependent calcium channels (VDCC), and lead to mitochondrial calcium overload by opening mitochondrial calcium channels and activating mitochondrial calcium uniporter (MCU). In addition, the activation of VDCC by inhalational anesthetics also triggers the release of Ca^2+^ from the endoplasmic reticulum (ER) by activating IP3 or ryanodine receptors on the ER membrane. The increase in cytosolic calcium, on one hand, exacerbates neuroinflammation through CaN-mediated mitochondrial retrograde signaling, and on the other hand, exacerbates mitochondrial calcium overload. Mitochondrial calcium overload mediates the opening of the mitochondrial permeability transition pore (mPTP), leading to a decrease in mitochondrial membrane potential (MMP) and the release of cytochrome C, inducing mitochondrial dysfunction and neuronal apoptosis. Furthermore, calcium overload leads to increased ROS production, activation of the NLRP3 inflammasome, and decreased ATP production, further exacerbating neuroinflammation and mitochondrial dysfunction

Mitochondrial calcium is crucial for the production of ATP [103]. However, calcium overload caused by anesthesia and surgery can lead to ATP imbalance, mitochondrial dysfunction, release of inflammatory factors, neuronal cell apoptosis, abnormal neurotransmitter release, and synaptic transmission disorders, ultimately promoting the occurrence of POCD [6, 11, 14, 28, 51] (Fig. 2). Studies have found that sevoflurane inhalation may induce mitochondrial mPTP opening, ROS increase, and reduction in ATP production by increasing intracellular calcium ion concentration, leading to mitochondrial dysfunction and structural damage [6]. Sevoflurane elevates intracellular Ca^2+^, leading to mitochondrial damage and subsequent mitochondrial-mediated apoptosis in the hippocampal neurons [6]. Isoflurane may induce neuronal apoptosis via calcium overload-mediated N-methyl D-aspartate receptor (NMDA-R) antagonism [104, 105]. Research by Liu et al. shows that sirtuin 3 can prevent anesthesia/surgery-induced cognitive decline in aged mice through inhibiting mitochondrial damage and hippocampal neuroinflammation caused by calcium overload [23].

The calcium transport channels and proteins of mitochondrial membrane are crucial for maintaining mitochondrial calcium homeostasis [106]. The mitochondrial calcium uniporter (MCU), situated in the inner mitochondrial membrane, is the key complex for mitochondrial calcium uptake [107]. Studies have found that surgery and anesthesia lead to intercellular environmental disorders and increased MCU expression in aged rats, resulting in increased mitochondrial calcium uptake [29]. Mitochondrial calcium overload leads to increased ROS release and decreased MMP, thereby causing mitochondrial dysfunction [29]. However, administering the mitochondrial calcium absorption inhibitor Ru360 can reverse the above processes, maintain mitochondrial calcium homeostasis, and alleviate the occurrence of POCD [29].

Voltage-dependent calcium channels (VDCC) are key channels facilitating the influx of extracellular Ca^2+^. Studies have shown that isoflurane can activate L-type VDCC through GABAA receptors [108], while sevoflurane can affect VDCC by activating CDK5 [109, 110]. Activation of VDCC promotes the influx of Ca^2+^ into neurons and exacerbates mitochondrial calcium overload. Furthermore, VDCC activation induces Ca^2+^ release from the ER by stimulating IP3 or ryanodine receptors on the ER membrane [28, 111]. On one hand, increased cytochrome C release caused by mitochondrial calcium overload inhibits the negative feedback effect of cytoplasmic Ca^2+^ on IP3 receptors, while on the other, it activates caspase3 to cleave IP3 receptors, hence forming a vicious cycle of massive calcium release from the ER[28]. Research indicates that isoflurane can trigger increased Ca^2+^ release from the ER, potentially causing ER calcium depletion, protein synthesis inhibition, and severe cytotoxic reactions [6, 51, 112].

Isoflurane-induced Ca^2+^ escape from the ER leads to an increase in cytosolic Ca^2+^, which may activate mitochondrial retrograde signaling through the calcineurin (CaN) pathway [113]. This signaling pathway is an adaptive mechanism that activates the expression of nuclear genes such as *NF-KB* by transmitting dysfunctional mitochondrial signals [114], thereby triggering neuroinflammation and cognitive impairment.

Cyclophilin D (CypD), located in the mitochondrial matrix, is essential for mitochondrial function by regulating mPTP opening and maintaining MMP [115–117]. Sevoflurane can elevate CypD levels by diminishing the interaction between CypD and adenine nucleotide translocase (ANT), a constituent of mPTP [118]. Increased CypD levels compromise the integrity of mitochondrial membranes and disturb calcium ion homeostasis, thereby causing mitochondrial dysfunction and neurodevelopmental impairments, culminating in cognitive impairments in young mice [118–120]. The absence of CypD can mitigate sevoflurane-induced negative neurological effects [118, 121]. However, some studies have also found that the effect of CypD on neurons is bidirectional [122]. CypD-mediated transient mPTP opening regulates dendritic calcium dynamics by enhancing mitochondrial calcium release and downstream signaling, thereby promoting activity-induced dendritic outgrowth [122]. However, prolonged opening of the mPTP increases the release of cytochrome C, leading to neuronal apoptosis [122].

### Mitochondrial iron homeostasis imbalance

**Fig. 3:**
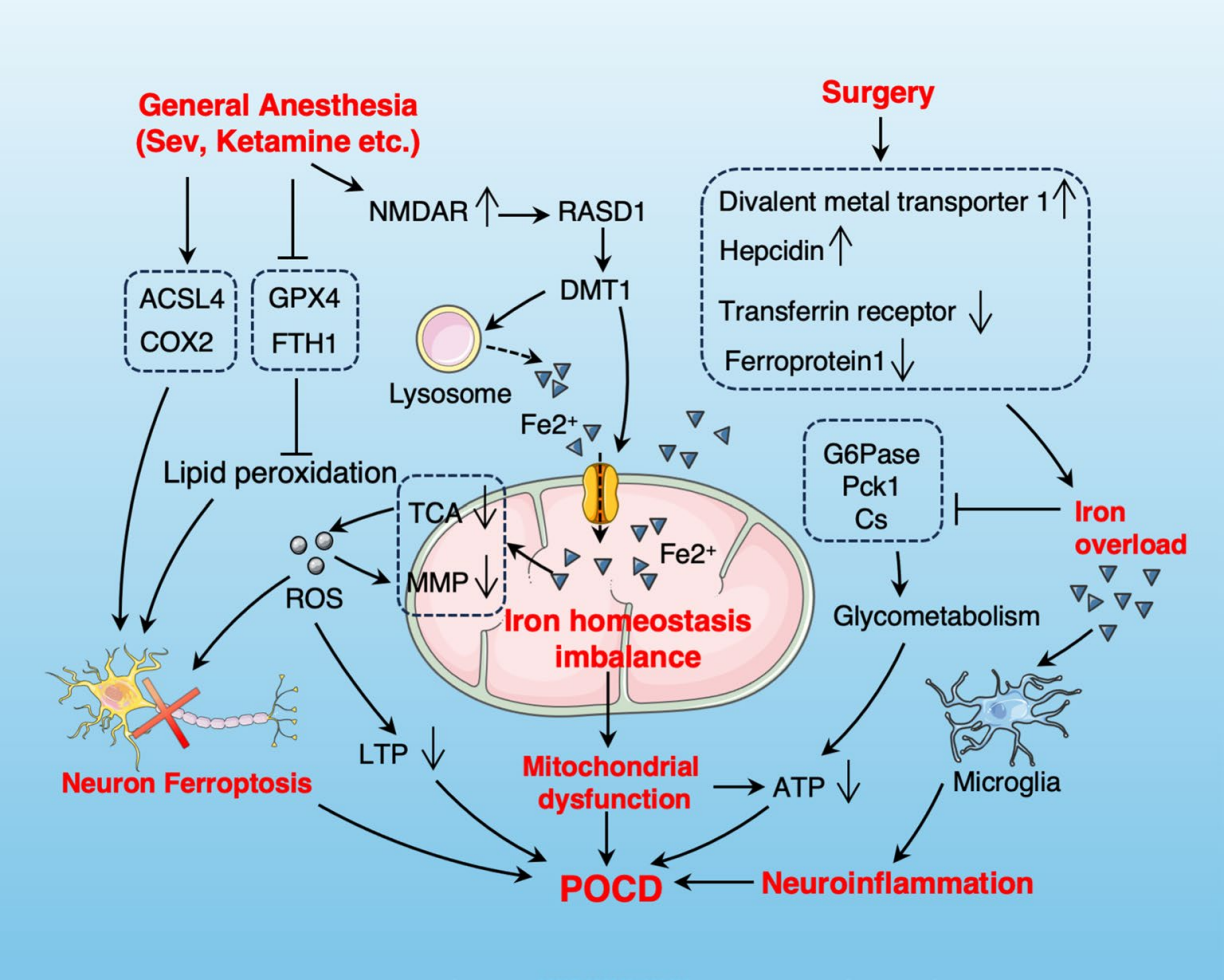
The role and mechanism of mitochondrial iron homeostasis imbalance in POCD. General anesthesia upregulates the expression of NMDAR, leading to mitochondrial iron overload by enhancing DMT1-mediated iron uptake and lysosomal iron release. Surgery can increase divalent metal transporter 1 and hepcidin, and decrease transferrin receptor and ferroportin 1, thereby causing iron overload. Mitochondrial iron homeostasis imbalance leads to reduced ATP production, increased ROS production through TCA and glucose metabolism pathways, and ultimately leads to mitochondrial dysfunction and POCD. Iron overload can also lead to pro-inflammatory activation of microglia, exacerbating neuroinflammation. In addition, iron metabolism abnormalities caused by general anesthesia can lead to the occurrence of POCD by promoting neuronal ferroptosis

Iron is crucial for normal neurological function, and neurons are particularly susceptible to changes in iron content [123]. Disruption of iron homeostasis can lead to significant neurotoxicity and neurogenetic abnormalities, interfere with neurotransmitter synthesis and release, and mitochondrial dysfunction [12]. Excess iron in the brain is linked to neurodegenerative diseases [12, 124] and can affect behavior and mood, resulting in learning and memory deficits [125, 126]. Iron is essential for energy production in glycolysis and the TCA cycle and serves as a cofactor for certain electron transport complexes in the mitochondrial respiratory chain [12, 35, 127, 128]. Intracellular iron homeostasis is important for maintaining normal mitochondrial function and glucose metabolism [35, 129, 130]. Prolonged or repeated exposure to general anesthesia can cause iron deposition in the hippocampus, cortex, and basal ganglia, potentially impairing learning, memory, and long-term potentiation in the hippocampus [128, 131]. Multiple studies found that sevoflurane treatment led to an abnormal increase in iron content (iron overload) in brain tissue [30, 35, 128]. Ketamine or sevoflurane can upregulate NMDA-R expression [132–134] and activate the GTPase RASD1, which interacts with the iron transporter divalent metal transporter 1 (DMT1) to enhance iron uptake and lysosomal release [128, 135]. This may be one of the mechanisms by which general anesthesia causes iron overload (Fig. 3). Surgery can elevate DMT1 and hepcidin levels while reducing transferrin receptor and ferroprotein 1, resulting in iron overload in the rat hippocampus [131] (Fig. 3). Excessive iron induces oxidative stress and impairs mitochondrial function while also disrupting glucose metabolism by inhibiting the expression of G6Pase, Pck1, and Cs [35, 131]. Sevoflurane-induced disruptions in iron and glucose metabolism contribute to POCD by decreasing ATP production and increasing neuronal apoptosis [35] (Fig. 3).

In addition, abnormalities in iron metabolism may lead to the occurrence of POCD through the mechanism of ferroptosis [30, 31, 128] (Fig. 3). Bioinformatics analysis and related studies have shown that mitochondrial-related ferroptosis may lead to cognitive deficits after sevoflurane administration [30]. Sevoflurane-induced ferroptosis involves not only the mechanism of iron overload but also the regulatory effect on key ferroptosis proteins such as ACSL4 and GPX4 [31]. During ferroptosis, mitochondria show reduced volume, increased membrane density, diminished cristae, and outer membrane rupture [31]. An in vitro study found that pretreatment with the selective ferroptosis inhibitor ferrostatin-1 preserved mitochondrial function and decreased neuronal cell death caused by isoflurane exposure, indicating that ferroptosis may contribute to isoflurane neurotoxicity [136]. A previous study found that ketamine or sevoflurane induces neuronal death, characterized by ferroptotic biomarkers including iron dependence, elevated lipid peroxidation, and reduced glutathione levels [137]. Administration of iron chelators can mitigate mitochondrial dysfunction, ferroptosis, and cognitive impairment caused by surgery and general anesthesia [128, 131]. We propose that general anesthesia-induced neurotoxicity and POCD are linked to disrupted iron homeostasis and iron-dependent ferroptosis.

### The effect of Tau protein on mitochondrial function

Abnormally phosphorylated Tau protein, serving as a characteristic marker of Alzheimer's Disease (AD), exerts a detrimental effect on cognitive function [138]. Research indicates that sevoflurane enhances tau protein phosphorylation [40], and hyperphosphorylated tau can damage mitochondrial function in the following ways (Fig. 1): (1) Interacting with MFN1, MFN2, OPA1 and DLP1, thus affecting mitochondrial dynamics. It primarily shifts the mitochondrial fission–fusion balance towards increased fission [139, 140]. (2) Inhibiting JIP1 and activating PP1 and GSK3 to prevent mitochondrial transport along microtubules [141, 142]. (3) Inhibiting mitochondrial respiratory chain complexes, causing oxidative phosphorylation dysfunction and increased ROS production [143–146]. (4) Interacting with VDAC1 to influence mPTP opening and closing, thus impairing membrane permeability [147].

### Age susceptibility to mitochondrial dysfunction

Anesthesia exposure may affect mitochondrial morphology and function in an age-dependent manner [5, 13, 32]. Studies have found that mitochondrial function in aged mice and neonatal mice is more susceptible to anesthesia, thereby becoming an important mechanism for neuron death and cognitive dysfunction [5, 14, 45]. Some studies argue that age susceptibility to general anesthesia may refer to neuronal age rather than biological age [26].

Exposure to sevoflurane during development impairs mitochondria and leads to cognitive deficits in neonatal rodents [148]. Early general anesthesia induces significant disturbances in mitochondrial morphology and function and inhibitory synaptic transmission during the peak period of synaptic development in developing rat brains [5, 13]. Exposure to sevoflurane during the neonatal period produces long-term and dose-dependent ultrastructural damage, including synaptic loss, reduced presynaptic mitochondrial localization, and altered postsynaptic density (PSD) length distribution [45]. Sevoflurane exposure mediates mitochondrial functional changes in the developing brain by activating the mtUPR, leading to changes in excitatory synaptic transmission [32]. Therefore, mitochondria may be an important early target of neuronal development and synaptic damage induced by general anesthesia [5, 13]. Kaley et al. found that limited early anesthesia exposure may induce persistent cellular dysfunction by inducing a state of sustained energy deficiency in mitochondria, leading to persistent neuroinflammation and protein toxicity, similar to the manifestations of chronic neurodegenerative diseases [36]. Sevoflurane exposure during infancy may affect mitochondrial QC and regeneration pathways, leading to the persistence of fragmented and energy-poor mitochondria, adversely affecting mitochondrial protein homeostasis and oxidative phosphorylation in adulthood, hence inducing permanent neuron dysfunction [36]. In addition, early changes in mitochondrial transport or fission may make surviving synapses more sensitive to subsequent anesthesia and surgical exposure [45]. However, one study also found that changes in mitochondrial function and excitatory/inhibitory synaptic transmission imbalance caused by sevoflurane exposure are transient and do not cause long-term behavioral changes [149].

Older individuals show heightened mitochondrial senescence, significantly contributing to various aging-related disease mechanisms [150]. The reduction of cytochrome C may be an important factor in the aging-induced decline of mitochondrial oxidative phosphorylation capacity, which affects ATP production in older brains [150]. In addition, the brains of older subjects exhibits increased mitochondrial basal oxidative stress, and neurons are highly exposed to ROS products [66]. However, studies on aged rodents have shown that anesthesia induces extensive formation of dendritic spines during critical synaptogenesis, rather than extensive neuronal apoptosis [151–153].

## The effect of mitochondrial oxidative stress on POCD

**Fig. 4:**
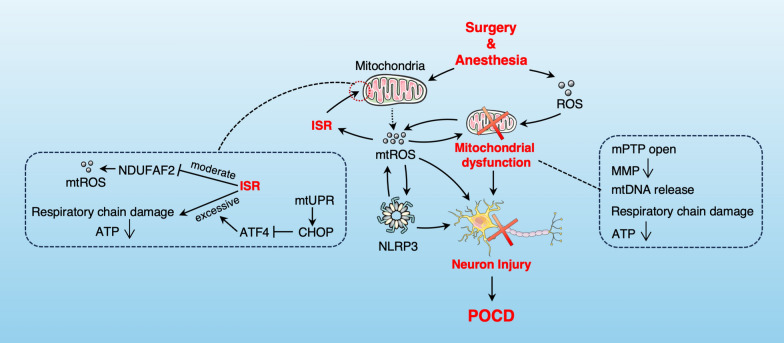
The role and mechanism of mitochondrial oxidative stress in POCD. After surgical and anesthetic exposure, mitochondria are not only the main source of ROS generation but also the main target of oxidative damage. Oxidative damage leads to mitochondrial dysfunction, such as decreased mitochondrial membrane potential (MMP), mtDNA release, impaired respiratory chain, and reduced ATP production. In addition, the activation of the integrated stress response (ISR) regulates oxidative stress through mitochondria, and excessive ISR can lead to mitochondrial energy imbalance and respiratory chain dysfunction, further increasing the release of mtROS. The increased production of mtROS exacerbates neuronal damage and the occurrence of POCD through a crosstalk with neuroinflammation

Neurons are particularly susceptible to ROS and reactive nitrogen species (RNS) damage owing to their high metabolic rate, fatty acids prone to peroxidation, abundant transition metals that catalyze ROS formation, and low antioxidant levels [154, 155]. Increased oxidative stress leads to worsening mitochondrial dysfunction, hippocampal neuronal damage, and synaptic loss, resulting in learning and cognitive dysfunction [14, 23]. Under anesthesia and surgical exposure, mitochondria serve as both the primary source and target of ROS, leading to reduced efficiency in the mitochondrial respiratory chain and ATP production [156, 157], increased mtDNA mutations [158], and further ROS production, creating a self-perpetuating cycle of oxidative damage [14, 159] (Fig. 4). In addition, the interaction between mitochondrial oxidative stress and neuroinflammation further aggravates POCD [160, 161] (Fig. 4). mtROS contributes to NLRP3 inflammasome activation, while the resulting inflammatory response causes mitochondrial damage and mtDNA release, further elevating mtROS production [162]. The mitochondrial-targeted antioxidant SS-31 can inhibit NLRP3 inflammasome activation and alleviate isoflurane-induced cognitive impairment by blocking mtROS [163].

Anesthesia and surgery significantly elevate MDA activity and reduce SOD activity, indicating increased mitochondrial oxidative stress [23], which is associated with POCD induction [14]. Sevoflurane exposure leads to an upregulation of CypD, which results in the opening of mPTP and a decrease in MMP, while also leading to a decrease in the ratio of GSH/GSSG in the mitochondria, increasing oxidative stress, and ultimately leading to cognitive impairment in mice [40, 122, 164].

Telomerase is an enzyme responsible for extending the ends of chromosomes, and it is also closely related to delaying the aging process [165]. The catalytic subunit telomerase reverse transcriptase (TERT) of telomerase plays an important antioxidant function by translocating from the cell nucleus to mitochondria [166, 167]. Studies have found that a decrease in TERT levels in the aging brain leads to an increase in mtROS release [167]. In addition, surgical intervention also reduces telomerase activity and mitochondrial localization of TERT in the hippocampus [168]. Therefore, the aging brain is more susceptible to mitochondrial oxidative stress during the perioperative period and induces POCD.

The integrated stress response (ISR) is a signaling hub regulatory network induced by protein homeostasis imbalance, primarily achieved by controlling the rate of protein synthesis [169]. This regulation involves the function of the eukaryotic initiation factor 2 (eIF2) ternary complex [169]. The core reactions of the ISR involve eIF2α phosphorylation and elevated ATF4 expression, both of which are closely linked to oxidative stress and various neurodegenerative diseases [170–174]. Research indicates that mice with POCD exhibit notable oxidative stress damage and ISR activation in the hippocampus [169]. ISR has been shown to regulate oxidative stress levels through mitochondria [175]. Current research has found that ISR has a dual regulatory effect on mitochondria (Fig. 4). As one of the stress response mechanisms maintaining cellular homeostasis, moderate ISR selectively targets the mitochondrial complex I assembly factor NDUFAF2 for translational inhibition and reduces the production of ROS related to complex I [176]. Excessive ISR activation can cause mitochondrial energy imbalance and respiratory chain dysfunction [177]. C/EBP Homologous Protein (CHOP), a multifunctional transcription factor, rises early in mitochondrial dysfunction [178, 179] and interacts with C/EBPβ to inhibit ATF4 overexpression, thus mitigating excessive ISR activation [177]. Therefore, CHOP can serve as a means to balance ISR and mitochondrial oxidative stress, thus reducing the adverse effects of ISR [177].

## The effect of mitophagy on POCD

Mitophagy is a type of selective autophagy that serves as a key mitochondrial QC mechanism [42, 180, 181] and maintains mitochondrial homeostasis by isolating damaged or redundant mitochondria through autophagosomes and lysosomal degradation [181]. Mitochondrial damage can cause cellular energy production disorders, oxidative stress, and impaired signal transmission [182–184]. To cope with changes in cellular energy status, mitophagy supports the high energy demand of neurons by maintaining and renewing a healthy and active mitochondrial pool through mitochondria QC [185]. Mitophagy alleviates cellular stress from oxidative damage and is crucial in neurodegenerative diseases and aging [21, 186]. Impaired mitophagy leads to synaptic dysfunction and cognitive deficits by increasing oxidative damage and cellular energy defects [187]. Mitophagy mechanisms are categorized into ubiquitin-dependent and ubiquitin-independent pathways [188]. The most important mechanism of the ubiquitin-dependent pathway is the PINK1/Parkin pathway [43, 188]. The ubiquitin-independent pathway mainly initiates mitophagy by directly binding LC3 with related proteins such as Nip3, bnip3, and FUNDC1 on the outer membrane of mitochondria [38, 43, 188]. Research also indicates crosstalk between the ISR pathway and activation of mTORC1 and AKT, with mitophagy partially mediated by ATF4 expression induced by eIF2a phosphorylation [189].

Research has found that sevoflurane-induced cognitive dysfunction in aged rats is associated with mitophagy dysfunction [21, 190]. Sevoflurane-induced SIRT1 expression reduction can induce cognitive dysfunction by inhibiting mitophagy and promoting activation of the inflammasome and cell apoptosis [191]. Sevoflurane causes the accumulation of damaged mitochondria, prevents the formation of autolysosomes, disrupts lysosomal acidification, and inhibits mitophagy flux [21], which may be related to inhibition of the Parkin pathway [190]. However, rapamycin treatment can reverse the loss of mature dendritic spines and improve sevoflurane-induced cognitive impairment by promoting mitophagy in hippocampal neurons [21]. Isoflurane anesthesia and abdominal surgery in rats significantly reduced hippocampal synapse-associated protein 25 (SNAP25) expression, impairing mitochondrial clearance and causing postoperative cognitive decline by inhibiting PINK1-mediated mitophagy [192]. Anesthesia and surgery-induced PINK1-mediated mitophagy defects activate caspase-3/GSDME-dependent neuronal pyroptosis, contributing to POCD [193]. Anesthesia and surgery-induced oxidative stress and impaired mitophagy flux jointly promote the release of mtDNA, thus becoming powerful promoters of NLRP3 inflammasome activation [194] and cGAS-STING pathway [195]. The cGAS-STING pathway is a crucial factor in chronic inflammation during aging, promoting pro-inflammatory microglial polarization and resulting in neurotoxicity and cognitive decline [196]. In AD patients, mitophagy flux in neurons and microglia were significantly impaired, resulting in abnormal autophagic vacuole accumulation, tau protein buildup, increased oxidative stress, synaptic dysfunction, neuronal loss, and cognitive decline [42, 197]. The accumulation of tau protein, in turn, disrupts mitophagy via the PINK1/Parkin pathway, creating a vicious cycle [198]. In addition to aged mice, studies have also shown that mitophagy dysfunction can lead to POCD after abdominal surgery in young mice[43].

## The cross talk of mitochondria and neuroinflammation in POCD

Alterations in mitochondrial metabolism and dysfunction are often observed in the early stages of neurodegenerative diseases [180]. The activation of mitochondria-dependent apoptotic pathways serves as the earliest warning signal for neuronal damage [5]. Mitochondrial dysfunction is an early trigger in isoflurane-induced neuronal damage [49]. Within the mitochondrial-neuroinflammation-POCD axis, mitochondria often occupy an upstream position and serve as one of the initiating factors, exacerbating neuroinflammation. Neuroinflammation serves as the central driving force behind POCD [3]. During stress induced by surgery and anesthesia, mitochondrial dysfunction often precedes the onset of neuroinflammation, and neuroinflammation, in turn, exacerbates mitochondrial dysfunction, thereby creating a vicious cycle [19, 98].

Mitochondria are considered the center of innate immune signaling pathways, including NLRP3 and cGAS/STING [194, 195, 199]. Under oxidative stress from surgery and anesthesia, increased mtROS production and mtDNA release from the open mPTP into the cytoplasm activate the NLRP3 inflammasome, exacerbating neuroinflammation and neuronal apoptosis, which ultimately results in POCD [19, 194]. In addition, mitochondrial SIRT3 promotes the occurrence of neuroinflammation by mediating oxidative stress responses [23]. Mitophagy defects lead to NLRP3 inflammasome activation via ROS accumulation, thereby contributing to POCD [43, 187, 194, 195]. Mitochondrial dysfunction, especially the decrease in MMP, may induce the activation of NF-κB by activating CaN [50]. Studies indicate that LPS stimulation induces mitochondrial dysfunction (reduced MMP) in astrocytes, triggers pyroptosis-related inflammatory factors via the STING/TBK-1 pathway, and promotes POCD occurrence [200]. Sevoflurane exposure leads to a decrease in mitochondrial oxidative phosphorylation function in the microglia, promoting the differentiation of microglia into pro-inflammatory A1 phenotype, thereby aggravating neuroinflammation [44]. Conversely, restoring mitophagy in microglia can exert neuroprotective effects by inhibiting neuroinflammation [201].

Primary neurons and SH-SY5Y cells were treated with tumor necrosis factor (TNF) [55]. After TNF exposure, the activity of CaN in primary neurons increased, promoting the activation of Drp1, leading to increased mitochondrial fragmentation and mitochondrial dysfunction [55]. CaN inhibitor FK506 has a function independent of drp1 and can alleviate mitochondrial dysfunction [55]. Inflammatory factors can also mediate Drp1 phosphorylation by activating CDK1 [36] and GSK3β [33], thereby promoting mitochondrial fission. The resulting mitochondrial dysfunction further promotes the release of inflammatory factors, forming a vicious cycle [36].

## POCD therapy mediated by mitochondria

Given the crucial role of mitochondria in POCD, numerous studies have focused on mitochondrial-targeted treatments, yielding promising outcomes (Table 2).

**Table 2:** Beneficial treatment of POCD by targeting mitochondria

Treatment	Mechanisms	References
Dexmedetomidine	Inhibit mitochondrial oxidative stress, promote mitophagy, activate mitoKATP	[159, 202–205]
Luteoloside	Increase ATP production, restore MMP and mitochondrial dynamics	[34]
Methylene blue	Inhibit mitochondrial fission	[87]
Heme	Inhibit mitochondrial damage and apoptosis, improve mitochondrial dynamics	[86]
Mdivi-1	Inhibit mitochondrial fission	[88]
Pramipexole	Protecting mitochondrial integrity	[206]
Rehabilitative resistance exercise	Improve mitochondrial biogenesis and dynamics	[98]
SIRT3	Inhibit mitochondrial oxidative stress	[23]
SESN1	Promote mitophagy, inhibit mitochondrial oxidative stress	[207, 208]
SS-31	Promote mitophagy, inhibit mitochondrial oxidative stress	[195]
Electroacupuncture	Inhibit mitochondrial oxidative stress (protect TERT)	[168]
Ru360	Reduce mitochondrial calcium overload	[29]
Melatonin	Alleviate mitochondrial dysfunction (upregulate CypD)	[122]
Deferoxamine	Reduce mitochondrial iron accumulation	[131]
Honokiol	Promote mitophagy	[43]
Varenicline	Promote mitophagy	[85]
Lidocaine	Restore MMP	[49]
Esketamine	Inhibit mitochondrial depolarization	[200]

### Dexmedetomidine

PGC-1α plays an important regulatory role in cellular metabolism and mitochondrial biosynthesis [96, 97, 209]. The absence of PGC-1α can lead to mitochondrial dysfunction and oxidative stress [210]. Dexmedetomidine ameliorates brain damage and neurological deficits in the intracerebral hemorrhage (ICH) model by suppressing oxidative stress resulting from the deactivation of the PGC-1α pathway and mitochondrial dysfunction [159]. Dexmedetomidine treatment enhances outcomes in sevoflurane-induced neurotoxicity and POCD by enhancing mitochondrial autophagy and mitigating oxidative stress in the mitochondria [202–204]. Dexmedetomidine exerts a neuroprotective effect in models of ischemia or tissue hypoxia by activating mitochondrial ATP-sensitive potassium channels [205].

### Improvement of mitochondrial occurrence and dynamics

Luteoloside enhances ATP production and MMP recovery, reversing the mitochondrial dynamics disorder induced by sevoflurane, thus mitigating the incidence of POCD in aged rats exposed to sevoflurane [34]. Methylene blue mitigates cognitive dysfunction induced by sevoflurane in aging mice by suppressing Drp1 SUMOylation, thus reducing mitochondrial fission [87]. Heme ameliorates mitochondrial damage and apoptosis triggered by sevoflurane exposure, as well as mitochondrial dynamics dysfunction. This protective effect may be associated with elevated neuroglobin levels [86]. Mdivi-1, a mitochondrial fission inhibitor, preserves mitochondrial integrity and diminishes anesthesia-induced synaptic damage and neurotoxic effects [88]. Pramipexole prevents cognitive decline in rats post-early anesthesia by safeguarding mitochondrial integrity [206]. The PGC-1α/BDNF pathway is intimately linked to mitochondrial biogenesis and dynamics and is pivotal in the formation and maintenance of dendritic spines and synapses within the hippocampus [211]. Rehabilitative resistance exercise activates hippocampal PGC-1α/BDNF/Akt/GSK-3β signaling, enhancing mitochondrial biogenesis and improving mitochondrial dynamics in post-operative aged mice [98].

### Anti-mitochondrial oxidative stress

SIRT3 is a crucial antioxidant enzyme that mitigates cognitive decline associated with anesthesia and surgery by suppressing mitochondrial oxidative stress and neuroinflammation [23]. Sestrin1 (SESN1), a stress response protein, is crucial in mitigating oxidative stress and DNA damage. Overexpression of SESN1 significantly reduces cognitive dysfunction induced by sevoflurane anesthesia, enhances mitophagy, and suppresses inflammasome activation and mitochondrial dysfunction through the activation of SIRT1 [207, 208]. SS-31, a mitochondrial-targeted antioxidant, enhances phb2-mediated mitophagy to inhibit mtDNA release, blocking the cGAS-STING pathway and M1 microglial polarization, thereby exerting a neuroprotective effect against POCD [195]. Electroacupuncture preconditioning protects against POCD resulting from mitochondrial oxidative stress by preserving the function of TERT in aged mice [168].

### Reducing mitochondrial calcium and iron imbalance

Ru360 alleviates POCD in aged mice by inhibiting MCU-mediated mitochondrial calcium overload [29]. Melatonin inhibits the upregulation of CypD induced by sevoflurane in PV neurons, thereby mitigating mitochondrial dysfunction, hippocampal injury, and cognitive deficits in neonatal mice [122]. Deferoxamine pretreatment can reduce mitochondrial iron accumulation in the hippocampus, decrease microglial activation, and mitigate POCD in rats [131].

### Regulating mitophagy

Honokiol-mediated mitophagy ameliorates cognitive dysfunction following surgery/sevoflurane anesthesia by suppressing the activation of the hippocampal NLRP3 inflammasome [43]. Varenicline alleviates cognitive impairment in aged mice following laparotomy by bolstering mitophagy [197].

### Restoring MMP

Lidocaine effectively mitigates mitochondrial injury and the decline in MMP induced by isoflurane in rats, thereby reducing POCD [49]. Esketamine can inhibit mitochondrial depolarization in astrocytes and alleviate postoperative cognitive decline in aged rats [200].

## Conclusion and perspectives

Mitochondria serve as the key organelles for energy production and metabolic regulation within cells and are essential for sustaining normal physiological functions. Mitochondrial function is susceptible to stress from surgery and anesthesia, particularly in older and developing nervous system cells that are more likely to experience dysfunction. Mitochondrial metabolic alterations and functional abnormalities frequently occur in the early stages of disease pathogenesis and could serve as one of the initiating factors for POCD. In addition, mitochondrial abnormalities are extensively involved in pathological processes like neuroinflammation and oxidative stress, synergistically contributing to the onset of POCD. Hence, mitochondrial abnormalities could potentially serve as both the "initiator" and "accelerator" of POCD. General anesthesia causes a decline in neuronal ATP production by inhibiting mitochondrial respiration, reducing MMP, and inducing mitochondrial calcium overload. Anesthesia and surgery trigger neuronal dysfunction and death via various mechanisms, such as promoting mitochondrial fission, decreasing MMP, enhancing mitochondrial calcium overload and mitochondrial-mediated ferroptosis, augmenting mitochondrial oxidative stress, and impairing mitophagy flux. The reduction in mitochondrial ATP production and the disruption of calcium and iron homeostasis due to anesthesia and surgery also hinder synaptic transmission and neurotransmitter synthesis and release in neurons. Additionally, mitochondrial dysfunction extensively cascades with mechanisms like neuroinflammation and oxidative stress, forming a vicious cycle. Mitochondrial abnormalities emerge early in the pathological process of POCD and even become upstream factors that influence other mechanisms. This intricate regulatory network collectively contributes to the onset of POCD.

It is noteworthy that many of the studies cited in the review are based on animal and cellular experiments, or even studies that utilize extracted mitochondria independent of organisms and cells. The applicability of these findings to human patients remains uncertain, so we need to approach the research conclusions with caution. To gain a clearer understanding of whether mitochondrial damage and dysfunction induced by anesthesia and surgery truly serve as "initiators" and "accelerators" in POCD, more high-quality clinical research is required to corroborate these findings.

Considering the pivotal role of mitochondria in POCD, targeting mitochondrial dysfunction may emerge as a novel therapeutic approach for POCD. Developing drugs that safeguard mitochondrial DNA, enhance MMP, and curtail ROS production can effectively enhance mitochondrial function, thus mitigating POCD symptoms. Future research should focus on developing drugs or treatments that stimulate mitophagy, aiming to enhance the homeostasis and functionality of nerve cells. POCD represents a complex pathological process; therefore, future studies should encourage interdisciplinary collaboration and employ a variety of technical approaches to seek comprehensive treatment strategies for POCD. Advancements in genomics and precision medicine will enable the creation of personalized treatment plans tailored to a patient’s genetic makeup, mitochondrial function, and other relevant factors.

## Data Availability

No datasets were generated or analysed during the current study.

## Abbreviations

POCD: Postoperative cognitive dysfunction
CNS: Central nervous system
ROS: Reactive oxygen species
MDA: Malondialdehyde
SOD: Superoxide dismutase
SP1: Specific protein 1
ER: Endoplasmic reticulum
IP3: Inositol 1, 4, 5-trisphosphate
mPTP: Mitochondrial permeability transition pore
MMP: Mitochondrial membrane potential
SNAP25: Synapse-associated protein 25
ETC: Electron transport chain
mtDNA: Mitochondrial DNA
TOM: Translocase of outer membrane
QC: Quality control
mtUPR: Mitochondrial unfolded protein response
UPS: Ubiquitin–proteasome system
MDV: Mitochondrial-derived vesicle
CaMKII: Calmodulin-dependent protein kinase II
OCR: OXYGEN consumption rate
CoQ: COENZYME Q
Fis1: Fission 1
Drp1: Dynamin-related protein 1
SUMO: Small ubiquitin-like modifier
SENP3: SUMO-specific protease 3
mitoKATP: Mitochondrial ATP-regulated potassium channel
NMDA-R: N-methyl D-aspartate receptor
MCU: Mitochondrial calcium uniporter
VDCC: Voltage-dependent calcium channels
CaN: Calcineurin
CypD: Cyclophilin D
ANT: Adenine nucleotide translocase
DMT1: Divalent metal transporter 1
PSD: Postsynaptic density
TERT: Telomerase reverse transcriptase
ISR: Integrated stress response
eIF2: Eukaryotic initiation factor 2
CHOP: C/EBP Homologous Protein
TNF: Tumor necrosis factor
ICH: Intracerebral hemorrhage
